# Health Risks Posed by Social and Linguistic Isolation in Older Korean Americans

**DOI:** 10.1093/geroni/igab046.1175

**Published:** 2021-12-17

**Authors:** Juyoung Park, Yuri Jang, Hyunwoo Yoon, Nan Sook Park, David Chiriboga, Miyong Kim

**Affiliations:** 1 University of Southern California, Los Angeles, California, United States; 2 School of Social Work, Portland State University, Portland, Oregon, United States; 3 University of South Florida, Tampa, Florida, United States; 4 School of Nursing, The University of Texas at Austin, Austin, Texas, United States

## Abstract

Guided by the double jeopardy hypothesis, the present study examined the health risks posed by the coexistence of social and linguistic isolation in older Korean Americans. Using data from the Study of Older Korean Americans (SOKA, n = 2,032), comparisons of four isolation typologies (no isolation, social isolation only, linguistic isolation only, and dual isolation) were made, and their impacts on physical (self-rated health), mental (mental distress), and cognitive health (cognitive performance) were examined. The ‘dual isolation’ group exhibited greater sociodemographic and health disadvantages. The odds of having fair/poor health, mental distress, and cognitive impairment were 2.21-3.17 times higher in the ‘dual isolation’ group than those in the group with no isolation. Our findings confirm that both social relationships and language proficiency are key elements for older immigrants’ social connectedness and integration, deprivation of which puts them at risk in multidimensions of health.

